# The stepwise rise of angiosperm‐dominated terrestrial ecosystems

**DOI:** 10.1111/brv.70039

**Published:** 2025-05-30

**Authors:** Wenna Ding, Daniele Silvestro, Renske E. Onstein, Mengxiao Wu, Zhekun Zhou, Yaowu Xing

**Affiliations:** ^1^ State Key Laboratory of Plant Diversity and Specialty Crops Xishuangbanna Tropical Botanical Garden, Chinese Academy of Sciences Mengla 666303 China; ^2^ Swiss Federal Institute for Forest, Snow and Landscape Research WSL Zürcherstrasse 111 Birmensdorf 8903 Switzerland; ^3^ Department of Biosystems Science and Engineering Federal Institute of Technology (ETH) Zurich Zürich Switzerland; ^4^ Gothenburg Global Biodiversity Centre University of Gothenburg Gothenburg 413 19 Sweden; ^5^ Department of Biological and Environmental Sciences University of Gothenburg Gothenburg 405 30 Sweden; ^6^ Swiss Institute of Bioinformatics Basel 4056 Switzerland; ^7^ Naturalis Biodiversity Center Darwinweg 2 Leiden 2333CR the Netherlands; ^8^ German Centre for Integrative Biodiversity Research (iDiv) Halle‐Jena‐Leipzig Puschstraße 4 Leipzig 04103 Germany

**Keywords:** flowering plants, angiosperm‐dominated biome, ecological dominance, floristic turnover, modernization, latitudinal biodiversity, terrestrial ecosystems

## Abstract

Angiosperms are the most diverse and abundant plant taxon today and dominate the majority of Earth's terrestrial ecosystems. They underwent rapid divergence and biogeographic expansion from the early to the middle Cretaceous. Yet, transformative ecosystem change brought about by the increased ecological dominance of angiosperms unfolded progressively until the Late Cretaceous. After the Cretaceous–Paleogene (K–Pg) boundary, angiosperms restructured terrestrial ecosystems towards a modern form. By the Neogene, crown groups that make up modern terrestrial angiosperm biodiversity radiated, and regional floristic distinctions were established concurrently with the steepened latitudinal and vertical temperature gradients. Here, we summarize, based on fossils and molecular evidence, when and how angiosperms came to diversify, dominate, and shape terrestrial ecosystems, leading to the emergence and spread of angiosperm‐dominated floras. We highlight five major phases of angiosperm evolution that took place against a background of palaeogeography and climate changes. There is a consistent delay in ecological dominance after lineage origination and taxonomic diversification, as a result of which angiosperms did not achieve ecological dominance across terrestrial biomes in a single step. The patterns of diversity seen among extant angiosperms, the dominant angiosperm groups within modern ecosystems, and the restriction of different groups of angiosperms to different parts of the world, reflect the contingent nature of the process of lineage diversification in the context of long‐term, substantial and ongoing environmental change. Determining the origins, diversification, and ecological dominance of angiosperms continues to be a challenge and requires elucidation of their early forms, functions, habitats, and environmental interactions throughout evolutionary history.

## I. INTRODUCTION

Angiosperms, known as flowering plants, are central to Earth's terrestrial ecosystems and exhibit remarkable ecological dominance across most of the world's major biomes with the exception of boreal and montane conifer forests (Folk, Siniscalchi & Soltis, 2020). Their ecological and floristic prominence in today's ecosystems can be recognized by their high biomass (or abundance) and number of species, positioning them as keystone taxa that disproportionately influence community assembly, trophic dynamics, and biogeochemical cycling processes (Benton, Wilf & Sauquet, 2022). As the most species‐rich plant group, angiosperms account for about 90% of all known extant plant diversity with more than 350,000 described species and possibly many still unknown to science (Antonelli *et al*., 2023; Qian, Zhang & Zhao, 2022). The rise to dominance of flowering plants changed land plant communities irreversibly, contrasting with earlier terrestrial ecosystems in which other plant groups – such as gymnosperms and ferns – dominated (Condamine *et al*., 2020; Benton, Wilf & Sauquet, 2022; Silvestro *et al*., 2015). It brought novel ecological features and functions, leading to the transformation of terrestrial ecosystems into modern forms. For example, tropical rainforests with intricate structures shaped by angiosperm trees functioning as ecosystem engineers created new conditions for the radiation of other land plants, in which ferns (Schneider *et al*., 2004), lianas (Collinson & Hooker, 2003), and epiphytes diversified (Xiang *et al*., 2016), and upon which other organisms have come to rely (Friis, Crane & Pedersen, 2024; Chomicki & Renner, 2017). The ecological interactions between flowering plants and their pollinators (Peris & Condamine, 2024; Asar, Ho & Sauquet, 2022; Peña‐Kairath *et al*., 2023), dispersers (Eriksson, Friis & Löfgren, 2000; Onstein, Kissling & Linder, 2022), and herbivores have built one of the major features of the trophic web in nature (Charles‐Dominique *et al*., 2016). Moreover, key trait innovations and their combination, such as double fertilization, pollen tube competition, carpels, xylem vessels, and reticulate‐veined leaves distinguish angiosperms from other plants (Wing & Boucher, 1998; Onstein, 2020; Leslie, Simpson & Mander, 2021; de Boer *et al*., 2012; Feild & Arens, 2005). The far‐reaching influence of the expansion of angiosperms during the Late Cretaceous and early Paleogene has been termed the Angiosperm Terrestrial Revolution (ATR) (Benton *et al*., 2022; Barba‐Montoya *et al*., 2018). However, transformation did not take place synchronously across different biomes, with many developing much later than the ATR and throughout the Cenozoic. The continuous change and diversification of flowering plants during the Cenozoic significantly influenced the evolution of terrestrial biomes on a global scale. Therefore, understanding how angiosperms differed from previous vegetation and how they evolved into modern forms is vital for interpreting their impacts on ecosystem evolution and our capacity to link these to physical global change.

In this review, we integrate the plant fossil record and phylogenetic evidence to understand the origination, development, and establishment of major modern‐type angiosperm‐dominated floras within their historical environmental context. We identify five major phases of angiosperm evolution and characterize the transformations leading to the emergence of modern angiosperm‐dominated floras. This transformation is marked by floristic regionalization and the emergence of extant dominant groups that replace previous ones in a geographical and changing climatic context (Silva de Miranda *et al*., 2018; Jaramillo, 2023; Donoghue & Edwards, 2014; Woodward, Lomas & Kelly, 2004; Crisp *et al*., 2009; Crisp, 2006). This review provides a context for how angiosperms assembled into modern biomes and holds insight into the origin and maintenance of biodiversity through time.

## II. PHASE 1: THE HIDDEN DIVERSITY OF ANGIOSPERMS DURING THE EARLY CRETACEOUS

The Early Cretaceous, spanning from *ca*.145 million years ago (Ma) to 100 Ma, marked a significant evolutionary milestone when flowering plants began to appear, diversify, and spread in the fossil record, laying the foundation for the vast variety of plant life that dominates the Earth today. This period saw the emergence of various angiosperm lineages, revealing a hidden diversity that has only recently come to light through fossil evidence and molecular analyses.

The earliest unequivocal fossilized angiosperms are from the Valanginian–Hauterivian (~135 Ma) with scattered occurrences of pollen grains from Israel, Morocco, Italy and England (Coiro, Doyle & Hilton, 2019). The exact origin of flowering plants will precede these records but remains debated (Shi *et al*., 2021; Silvestro *et al*., 2021; Zuntini *et al*., 2024). In the succeeding Barremian and Aptian stages (∼125 to 113 Ma), angiosperms rapidly expanded their geographical range worldwide (e.g. Portugal, Spain, northeastern China, eastern North America, Australia, and Brazil) with exquisitely preserved angiosperm flowers, carpels, stamens with pollen sacs, fruits and seeds providing indisputable evidence (Coiro *et al*., 2019; Heimhofer & Hochuli, 2010; Jud, 2015; Crane, Friis & Pedersen, 1995; Friis, Pedersen & Crane, 2006a; Friis *et al*., 2015; Sun *et al*., 2002; Taylor & Hickey, 1990; Gomez *et al*., 2020). Critical assessments assigned some of these anatomically preserved reproductive structures to the early diverging ANA grade (Doyle & Upchurch, 2014; Friis, Crane & Pedersen, 2018a, 2019c), Chloranthales (Friis, Crane & Pedersen, 2019b), Magnoliales or magnoliids (Friis, Crane & Pedersen, 2019a), Laurales (Friis, Crane & Pedersen, 2017), or basal eudicots (Boukhamsin, Peyrot & Vecoli, 2022; Jud, 2015; Friis, Mendes & Pedersen, 2018b), with less common attributions for monocots (Coiffard *et al*., 2019). However, the systematic affinities of many early angiosperm fossils remain unclear. For example, Archaefructaceae, an extinct basal angiosperm family of herbaceous aquatic plants, originally was placed as sister to extant angiosperms (Sun *et al*., 2002), but later was repositioned among near‐basal eudicots (Friis *et al*., 2003). As an increasing number of extinct angiosperm lineages continue to be identified, including those with exotestal seeds and multipartite, apocarpous flowers featuring unique traits not seen among extant species (Friis, Crane & Pedersen, 2020, 2021; Friis *et al*., 2018a, 2019a), the previously underestimated diversity of flowering plants is now being recognized. Analysis of a comprehensive data set focusing on floral structure across angiosperms (López‐Martínez *et al*., 2024) suggested that they reached the highest floral disparity in the Early Cretaceous.

Despite the ongoing challenges in reconciling discrepancies between molecular and fossil evidence on the origin and crown age of angiosperms, both lines of evidence agree that angiosperms underwent a remarkable diversification during the late Varremian–Albian (Silvestro *et al*., 2015; Dimitrov *et al*., 2023; Crane *et al*., 1995; Silvestro *et al*., 2021; Lidgard & Crane, 1990). A Bayesian analysis of fossil records and extant diversity inferred a surge in lineage accumulation between 125 and 100 million years ago (Silvestro *et al*., 2021). Phylogenetic inference further suggests that over 80% of extant angiosperm orders originated during this early diversification burst (Zuntini *et al*., 2024).

As systematic studies of mesofossil assemblages have identified growing numbers of angiosperms compared to contemporaneous macrofossil and palynological records, it has been realized that angiosperms could be locally diverse during the late Barremian to early Albian, though they remained ecologically subordinate within most paleofloras (Friis *et al*., 2019c, 2022, 2024). For example, in one of the most extensively studied charcoalified mesofossil floras from the late Barremian to early Aptian Torres Vedras in Portugal, angiosperms (*ca*.150 spp.) accounted for approximately 62% of all identified species (Friis *et al*., 2019c), but constitute only about 18% of the mesofossil specimens (Friis *et al*., 2019c, 2024). Spore‐bearing plants and conifers were dominant in terms of abundance in the Torres Vedras flora. Although angiosperms were occasionally dominant both in species richness and abundance, such as in the mesofossil assemblage of Catefica from the Aptian–early Albian (Friis *et al*., 2022), the relative rarity or even absence of angiosperms in many other fossil floras reflects geographic variations of their diversity and palaeoenvironmental constraints during their early expansion (Friis *et al*., 2024). The high diversity of angiosperms in certain taphonomic conditions, such as floodplain habitats, braided river environments, or disturbed areas where fires were frequent, suggests that angiosperms were opportunistic colonizers that took advantage of disturbed ephemeral habitats in their early rising stage (Kvaček *et al*., 2024; Friis *et al*., 2024). Moreover, the observed underrepresentation of angiosperm pollen grains in palynological assemblages compared to mesofossil floras likely results from the relatively low stature of early angiosperms (Friis *et al*., 2024), the prevalence of entomophily limiting wind dispersal (Hu *et al*., 2008), and the poor preservation potential of some basal angiosperm pollen (e.g. Laurales) (Traverse, 2007), reflecting their restricted ecological niche during initial evolutionary phases. By the end of the Albian, angiosperms became locally abundant components of early successional vegetation and migrated polewards in Asia, North America, and the Antarctic (Kvaček *et al*., 2020; Golovneva *et al*., 2018).

The environmental conditions during early angiosperm radiation are crucial for understanding the selective pressures and habitat contexts that led to the evolution of key angiosperm features (Feild & Arens, 2005). While tectonic‐driven climate changes established suitable bioclimatic conditions for angiosperm expansion and diversification (Chaboureau *et al*., 2014), ecological interactions and adaptive traits that enabled them to thrive in diverse environments may have also contributed to their success in early successional environments and to their evolutionary success (Berendse & Scheffer, 2009; Brodribb & Feild, 2010; Feild & Arens, 2005). Yet, the role of ecological opportunities and key innovations in fuelling the early adaptive radiation of angiosperms remains unclear.

## III. PHASE 2: THE RISE TO ECOLOGICAL DOMINANCE AFTER THE LATE CRETACEOUS

By the Late Cretaceous, over half of the extant angiosperm families had originated, with stem ages for most (58–80%) families estimated at ~100 to 90 Ma (Silvestro *et al*., 2021; Ramírez‐Barahona, Sauquet & Magallón, 2020; Wing & Boucher, 1998; William, 2008). Core eudicots that comprise the majority of extant angiosperm species became increasingly diverse across a wide geographic range and gained prominence compared to other groups of angiosperms during this period (Friis, Pedersen & Crane, 2016; Barreda, Palazzesi & Olivero, 2019; Coiffard *et al*., 2012; Wing & Boucher, 1998; Friis *et al*., 2024; Gandolfo *et al*., 2018). Unequivocal palm fossils and many other monocots emerged from the Coniacian onwards [see review Friis *et al*. (2024) and references therein]. The marked increase in angiosperm diversity is closely linked to their evolution of reproductive and ecophysiological innovations (Leslie *et al*., 2021; William, 2008). Key floral traits evolved associated with specialized modes of insect pollination, including bilateral symmetry, prolonged calyx tubes, fused petals (sympetaly), and the production of nectar and resin rewards (Crepet, 1996; Bao *et al*., 2019). Additionally, by the Cenomanian, angiosperms had evolved highly efficient leaves with vein densities significantly surpassing those of non‐angiosperms, marking a major advancement in photosynthetic capacity and ecological competitiveness (Feild *et al*., 2011; Brodribb & Feild, 2010). These functional trait innovations likely acted synergistically, driving the rapid diversification and ecological dominance of angiosperms during the Late Cretaceous (Boyce & Leslie, 2012).

Angiosperms showed high taxonomic diversity over a broad geographic range from the Cenomanian (Coiffard *et al*., 2023; Upchurch & Dilcher, 1990; Wang & Dilcher, 2006), and became relatively abundant across a variety of habitats (Váchová & Kvaček, 2009; Kvaček *et al*., 2024; Coiffard *et al*., 2012; Halamski *et al*., 2020; Kvaček, 2017), reflecting the widening of their ecological niches. For example, widespread dominance of angiosperms in the vegetation of alluvial plains across much of Cenomanian Europe contrasts with Barremian–Albian times when angiosperms were known to occupy only disturbed habitats (Kvaček *et al*., 2024). In tropical regions, angiosperms dominated floristic diversity and even exceeded 90% of species composition, with a high representation of monocots in African Campanian assemblages (Coiffard *et al*., 2023). In comparison, contemporaneous mid‐latitude subtropical floras, exemplified by the Grünbach assemblage, exhibited lower angiosperm representation at 68% (Coiffard *et al*., 2023). Angiosperms did not attain ecological dominance in terms of abundance and biomass concurrently with their within‐flora diversity across broad geographic ranges. Instead, they largely shared ecological dominance or occupied subordinate roles with gymnosperms and free‐sporing plants (e.g. pteridophytes, lycopsids, ferns, sphenopsids) (Friis *et al*., 2024; Friis, Crane & Pedersen, 2011), displaying distinct biogeographic patterns of dominance across latitudinal gradients (Lupia, Lidgard & Crane, 1999). For example, in northern mid‐latitudes, quantitative studies of a mid‐Maastrichtian Wyoming macrofossil flora revealed that angiosperms had a subordinate role in these areas of fern‐dominated vegetation with a relatively low vegetation cover (12%), even though they constituted higher species richness (61%) (Wing, Hickey & Swisher, 1993). Similarly, analyses of the extensive North American palynological record from the Late Cretaceous indicate a ~ 10‐million‐year lag in palynological flora abundance relative to angiosperm dominance of floristic diversity (Lidgard & Crane, 1990). Angiosperms diversified and became abundant later at higher palaeolatitudes (Friis *et al*., 2011). In the Antarctic, angiosperms progressively increased within‐flora diversity at the expense of spore plants and gymnosperms, while their within‐flora diversity and abundance were consistently low compared to contemporaneous middle–low latitudes (Barreda *et al*., 2019; Lupia *et al*., 1999). Angiosperms then became the most diverse and abundant plant group in the Antarctic until mid‐Campanian times (*ca*. 78 Ma) (Barreda *et al*., 2019). Although angiosperms were predominant in Cenomanian mega‐ and mesofossil assemblages from low and middle latitudes, such as in central Europe (the Peruc flora) (Halamski *et al*., 2020; Kvaček *et al*., 2024), the USA (the Dakota flora) (Upchurch & Dilcher, 1990), and Egyptian macroflora (El Atfy *et al*., 2023), their corresponding microfossil floras were dominated in most cases by non‐angiosperm pollen or spores (Lidgard & Crane, 1990; Wing *et al*., 1993; Halamski *et al*., 2020; Halamski, Kvaček & Vajda, 2016). This discrepancy may result from the underrepresentation of certain lineages that constituted substantial parts of the vegetation, such as Laurales (Halamski *et al*., 2020; Kvaček *et al*., 2024) compared with the considerably more abundant propagules of wind‐dispersed gymnosperms and ferns in the palynological record (Halamski *et al*., 2020, 2016). Additionally, variations in the preservation potential of plant organs and depositional environments may skew the overall representation of plant communities among data sets (Slater & Wellman, 2015; El Atfy *et al*., 2023; Cleal *et al*., 2021). Despite the inherent bias, macrofossil and palynological assemblages capture different aspects of angiosperm evolution and diversity and their integration could provide a more comprehensive view of plant community dynamics over geological time (Cleal *et al*., 2021; Carvalho *et al*., 2021).

Fossilized angiosperm wood serves as an important proxy for determining the time at which angiosperms attained large stature and began to structure forest canopies. Angiosperm wood fossils were rare throughout the Early Cretaceous (Nunes *et al*., 2018; Philippe *et al*., 2008; Wheeler & Lehman, 2009), with trunks or sizeable wood fragments not documented until the Cenomanian (Gryc, Vavrčík & Sakala, 2009) and Turonian (Jud *et al*., 2018; Chin *et al*., 2019). By the Campanian and Maastrichtian, the increased occurrences and diversity of angiosperm wood indicates that a range of angiosperm lineages had attained large stature and the capacity to form angiosperm‐dominated forests (Friis *et al*., 2024). Determining angiosperm ecological dominance in the geological past demands evidence beyond taxonomic diversity within floras, incorporating both relative abundance data and additional proxies for a more comprehensive understanding. Another example is from the Maastrichtian Guaduas flora, Colombia. An open‐canopy structure dominated by gymnosperms (primarily Araucariaceae) has been inferred based on leaf vein length per area and stable carbon isotope ratios within individual taxa, despite angiosperms exhibiting the highest morphological diversity and co‐dominating in abundance with spore‐bearing plants in this Maastrichtian assemblage (Carvalho *et al*., 2021).

**Fig. 1:**
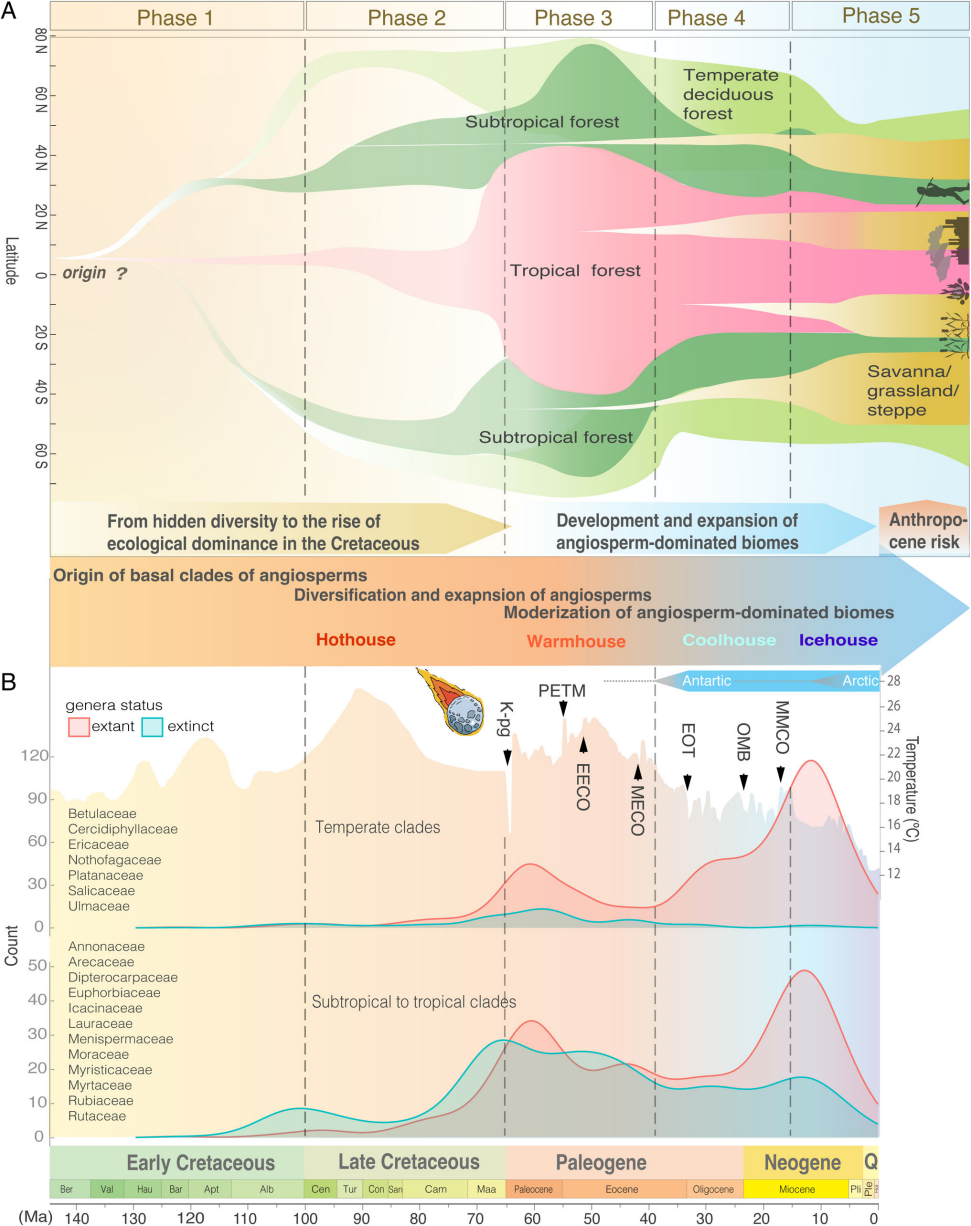
Stepwise evolution of angiosperms and angiosperm‐dominated biomes in Earth history during the Cretaceous and Cenozoic. (A) Conceptual evolution of representatives in angiosperm‐dominated biomes across time and space. The timeline of origin and geographic expansion of different biomes and their representative species is roughly drawn based on palaeobotanical evidence discussed in the main text. The question mark denotes that when and from where angiosperms originated remains unknown. The icons in phase 5 represent the major threats and habitat loss from human activities, fires, urbanization, and intensive agriculture across biomes generally. (B) Density of fossil records of several major clades in temperate and subtropical to tropical regions for angiosperm genera based on Silvestro *et al*. (2021) and the *Paleobiodb* database (https://paleobiodb.org/). Spatial bias of fossil records and systematic incompleteness were observed between temperate and tropical lineages, with richer fossils excavated in the temperate Northern Hemisphere. The red and blue distributions refer to extinct (blue) or extant (red) morphotypes or fossil species during each time period. The global average temperature curve is from Scotese *et al*. (2021). The blue bar indicates whether there were well‐developed ice sheets (full blue bar) or intermittent ice sheets (dashed grey line and triangles). K–Pg, Cretaceous–Paleogene boundary; PETM, Paleocene‐Eocene Thermal Maximum; EECO, Early Eocene Climatic Optimum; MECO, Middle Eocene Climatic Optimum; EOT, Eocene–Oligocene transition; OMB, Oligocene–Miocene Boundary; MMCO, Middle Miocene Climatic Optimum. Ber, Berriasian; Val, Valanginian; Hau, Hauterivian; Bar, Barremian; Apt, Aptian; Alb, Albian; Cen, Cenomanian; Tur, Turonian; Con, Coniacian; San, Santonian; Cam, Campanian; Maa, Maastrichtian; Pli, Pliocene; Ple, Pleistocene; Q, Quaternary.

The ecological radiation of angiosperms revealed in the early Late Cretaceous coincides with a progressive long‐term warming trend (Fig. 1B), fragmented continental configuration and the expansion of temperate biomes that culminated during the Cenomanian–Turonian at the expense of desert areas (Chaboureau *et al*., 2014; Miller *et al*., 2011). The progressive decrease of arid zones led to increased connectivity between humid tropical and warm temperate zones from the Cenomanian (Chaboureau *et al*., 2014). Many angiosperm fossil‐rich localities are known from this time under temperate climate, including in the polar region (Herman & Spicer, 2010; Zolina, Golovneva & Spicer, 2020; West, Reichgelt & Basinger, 2021). Despite angiosperms expanded into the northern polar region, these Arctic dicots exhibited characteristically large leaf sizes and a high proportion of entire margins, features consistent with growth under warmer and milder climates (Spicer *et al*., 2003), and coexisted with representatives of the older Mesozoic floras, including descendants of Jurassic ferns, cycads, and conifers (Herman & Spicer, 2010; Zolina *et al*., 2020). Thus, these plant assemblages were fundamentally different from modern temperate broadleaved forests. Normapolles‐producing angiosperms related to ancestral groups of core Fagales can be traced back to the Cenomanian (Friis, Pedersen & Schönenberger, 2006b). These *Normapolles*‐type angiosperms underwent radiation during a transient phase characterized by relatively drier climates and punctuated cooling at middle palaeolatitudes of the Northern Hemisphere during the Late Cretaceous (Friis *et al*., 2024; Heimhofer *et al*., 2018). They likely grew as small, non‐woody plants or shrubs in xerophytic savanna‐type vegetation under dry or seasonally dry climates (Heimhofer *et al*., 2018). This early ecological adaptation contrasts with that of their extant relatives in clades such as Fagaceae and Betulaceae, which dominate modern broad‐leaved temperate to subtropical forests as large woody trees forming closed‐canopy ecosystems. Collectively, these findings indicate that angiosperms underwent rapid radiation toward ecological dominance by the end of the Cretaceous and became important components in diverse floristic regions in an ancient, unacquainted appearance. However, the scarcity of quantitative data on elemental abundance and biomass for many floristic assemblages underscores the challenges inherent in reconstructing the evolutionary trajectory of angiosperms towards ecological dominance.

## IV. PHASE 3: K–PG TO MIDDLE EOCENE: DEVELOPMENT OF MODERN‐EQUIVALENT ANGIOSPERM‐DOMINANT TROPICAL FORESTS

At the Cretaceous–Paleogene (K–Pg) boundary, the Chicxulub bolide impact resulted in a chain of devastating environmental changes and abrupt biodiversity collapse (Schulte *et al*., 2010; Hull *et al*., 2020; Wilf, Carvalho & Stiles, 2023). It is estimated that approximately 75% of marine species and land animals went extinct during the K–Pg mass extinction (Wilf *et al*., 2023; Johnson, 1993). In contrast to the tremendous extinction of marine invertebrates, all non‐avian dinosaurs (Schulte *et al*., 2010; Hull *et al*., 2020), and a dramatic loss of specialized insect herbivores (Carvalho *et al*., 2021), the Chicxulub bolide impact on plants, especially for angiosperms, remains debated. The global synthesis of fossil plant records suggests that there was a minor impact on the extinction rates of major lineages (families and orders) (Cascales‐Miñana & Cleal, 2014; Silvestro *et al*., 2015). Recent analysis of large species‐level phylogenies suggests a constant extinction rate through geological time despite the uncertain estimates of extinction rate through phylogenies (Thompson & Ramírez‐Barahona, 2023), and a crown angiosperm radiation after the K–Pg (Ramírez‐Barahona *et al*., 2020). On the other hand, increasing palaeobotanical evidence on both palynological and macrofossil records has demonstrated that the Chicxulub asteroid had a substantial impact on plants, with more than 50% of species going extinct in well‐dated macrofloras in the Americas (see review in Wilf *et al*., 2023) and similar pattern also found in New Zealand, Europe and Antarctica [see review in Vajda & Bercovici (2014) and references therein]. However, the magnitude of species loss and recovery time is geographically heterogeneous, which reflects factors like the distance from the Chicxulub impact site and marine and latitudinal buffering of the impact of the resulting winter (Wilf *et al*., 2023). In contrast to large extinctions in western North America and the Neotropics, less severe extinctions occurred in the maritime areas of temperate Gondwana (Wilf *et al*., 2023), suggesting that the influence of the K–Pg extinction on land plants was geographically heterogeneous. The survival and flourishing of angiosperms across the K–Pg has been suggested to be related to whole‐genome duplications (WGDs) observed across numerous clades (Huang *et al*., 2022; Van de Peer *et al*., 2021; Zhang *et al*., 2022a), endowing them with enhanced capabilities for efficient energy and carbon capture [Blonder *et al*., 2014; Wu, Han & Jiao, 2020; see review in Benton *et al*. (2022) and references therein]. Moreover, the ability to form a frost‐tolerant seed bank associated with seed dormancy and recalcitrance is also hypothesized to be a major factor in angiosperm survival of the K–Pg impact winter (Berry, 2020). The high survival rate among deciduous versus evergreen plants that are more sensitive to low temperatures left a profound imprint on Northern Hemisphere Cenozoic vegetation (Wolfe & Upchurch, 1986).

Environmental disturbances around the K–Pg boundary were important for breaking the dominance of older persistent and competitive lineages, setting the stage for the modernization of angiosperm‐dominated biomes. Following the K–Pg event, an unequivocal Neotropical rainforest, dominated by angiosperms, developed in Colombia during the early Paleocene (Carvalho *et al*., 2021). Over a period of approximately 6 million years, it developed into a forest that resembled modern Neotropical rainforests, featuring a closed, multistratal canopy and a similar community composition, including the dominant tree families in modern Neotropical rainforests: Fabaceae, Moraceae, Annonaceae, Euphorbiaceae, Lauraceae, Sapotaceae, Menispermaceae, Anacardiaceae, Meliaceae, Malvaceae, and Arecaceae (Carvalho *et al*., 2021; see review in Jaramillo, 2023). A detailed sequential stratigraphic analysis from Colombia with a high‐resolution time calibration provides direct evidence of the rise of angiosperms to true ecological dominance after the K–Pg. Palaeobiogeographical analysis of pollen fossils from Africa and India combined with molecular data and fossil amber records suggest a tropical‐African origin of Dipterocarpaceae and its dispersal to India leading to range expansion of aseasonal dipterocarps on the Indian Plate during the Late Maastrichtian and Paleocene (Bansal *et al*., 2022; Khan *et al*., 2020). Maastrichtian leaf fossils discovered in Central India, showing affinities to the extant genus *Dipterocarpus*, provide compelling evidence for the presence of Dipterocarpaceae in India during the K–Pg transition (Khan *et al*., 2020). Dipterocarpaceae did not colonize Southeast Asia until the middle to late Eocene following the India–Asia collision (Dutta *et al*., 2011), marking its emergence as one of the dominant tree taxon in Asian tropical forests. An angiosperm‐dominated Afrotropical rainforest has been described in northeastern Africa dating to around 80 Ma and showing modern features in terms of relatively high species richness and taxonomic composition (Coiffard *et al*., 2023). This finding indicates that the modern tropical rainforest might have evolved asynchronously across different regions. However, when and how angiosperms came to dominate the tropical rainforest and when it assembled into a modern appearance remains incompletely understood due to the fragmentary and temporally discontinuous nature of the tropical fossil record.

The rapid increase in global CO_2_ during the Paleocene–Eocene Thermal Maximum (PETM) fuelled global temperatures, leading to a “hyper‐thermal” period that persisted into the middle Eocene, and a remarkably shallow latitudinal temperature gradient and low seasonality (Storey, Duncan & Swisher, 2007; Keating‐Bitonti *et al*., 2011). These greenhouse conditions allowed for the spatial widening of thermophilic floras (also known as boreotropical floras) into the high Arctic (70° N) in some coastal plains (Suan *et al*., 2017), with the occurrence of palms and other subtropical elements even extending as far as 80 °N (Willard *et al*., 2019) (Fig. 1A), reaching their maximal poleward during the Eocene thermal. During this period, both thermophilic and mesophytic angiosperms were spread across mid to high latitudes, leading to a high degree of taxonomic similarity between Eurasia and North America (Su *et al*., 2020; Tang *et al*., 2019; Manchester, 1999). For example, over 70 plant forms from central Tibet of the early middle Eocene flora shared similarities with contemporaneous floras in both North America and Europe (Su *et al*., 2020). In the Southern Hemisphere, paratropical/subtropical forests developed in the midlatitudes of South America and expanded as far as 60–70° S (Fig. 1A). This is exemplified by mesothermal‐megathermal rainforests in Patagonia (Wilf *et al*., 2003) and the highly diverse frost‐free with occasional frost‐tolerant elements, including palms and Bombacoideae in the Antarctic continent during the early Eocene (Pross *et al*., 2012). Coincidentally, a marked diversification of tropical/subtropical lineages and the increased complexity of the forest were observed during this warm period (Jaramillo *et al*., 2010; Burnham, 2015) [see review in Jaramillo (2023) and references therein; Fig. 1B].

## V. PHASE 4: THE LATE MIDDLE EOCENE TO THE MIDDLE MIOCENE: FLORISTIC TURNOVER IN MIDDLE TO HIGH LATITUDES

After the Mid‐Eocene Climatic Optimum, a secular global cooling trend that intensified at the Eocene–Oligocene Transition (EOT) led to enhanced temperature seasonality in northern high latitudes (Eldrett *et al*., 2009; Toumoulin *et al*., 2022). This climate change triggered substantial restructuring of floristic composition along latitudinal gradients. The once widespread boreotropical/paratropical forests that thrived during the early Eocene receded from middle–high latitudes, gradually being replaced by elements of temperate deciduous broadleaved forests (Kvaček *et al*., 2014; DeVore & Pigg, 2010; Mijarra *et al*., 2009).

In Europe, the diversity of broadleaved evergreen or semi‐evergreen elements such as Araceae, Icacinaceae, Lauraceae, Menispermaceae, Malvaceae, Theaceae, Moraceae and many other thermophilic lineages declined considerably from the middle Eocene onward (Mijarra *et al*., 2009; Teodoridis & Kvaček, 2015; Moraweck *et al*., 2019; Del Rio & De Franceschi, 2020). Conversely, deciduous taxa such as Rosaceae and Betulaceae exhibited increasing diversity during this period (Kunzmann & Walther, 2002; Moraweck *et al*., 2019). In North America, after the thermal maximum, the paleoclimate became cooler and drier from the middle to late Eocene and continued into the Oligocene (DeVore & Pigg, 2010). The Oligocene flora of Colorado evidenced an ecological transition toward a more modern composition, with the appearances of xeric shrubland and herbaceous taxa that characterize the Colorado area today, reflecting broader climatic changes characterized by cooling temperatures and reduced summer rainfall (Leopold & Zaborac‐Reed, 2019). Additionally, open‐habitat grasses underwent considerable taxonomic diversification by the earliest Oligocene but had not yet achieved ecological dominance, persisting primarily in the understory or within forest glades (Strömberg, 2005). Similarly, at high latitudes of the Southern Hemisphere, warm‐temperate forests in Patagonia, Antarctica, Australia, and New Zealand were gradually replaced by cool temperate *Nothofagus*‐dominated vegetation by the Oligocene (Brea *et al*., 2015; Thompson *et al*., 2022; Korasidis *et al*., 2019; Prebble *et al*., 2021).

### (1) Development of broadleaved deciduous forests in the Northern Hemisphere

Deciduous temperate forests are predominantly distributed at high latitudes in the Northern Hemisphere today, with their canopies consisting of at least two‐thirds deciduous broadleaf foliage (notophyll–mesophyll) and up to one‐third evergreen (typically needleleaf) cover (Keith *et al*., 2022). Temperate deciduous forest was assembled through a complex mixture of elements derived from various sources. Fossil evidence suggests that many of these elements originated from mesic Cretaceous–Paleocene ancestors within ancient polar broadleaved deciduous forests that evolved over a prolonged period at high northern paleolatitudes (Zolina *et al*., 2020; West *et al*., 2021; Wolfe, 1987b) and/or originated from upland regions (Wolfe, 1987a). In addition, multiple lineages within the widespread boreotropical forests adapted locally to develop deciduous habits and frost tolerance in response to the global cooling that prevailed from the late Eocene (Zhang *et al*., 2022b; Edwards *et al*., 2017). Studies of *Viburnum* as a model system through a combination of phylogenetic and phenological analyses showed multiple transitions in leaf habits, with clear evidence of deciduous species evolving in areas with prolonged annual freezing (Edwards *et al*., 2017). This highlights the role of environmental pressures in shaping evolutionary trajectories and ecological adaptations, ultimately leading to the widespread emergence of deciduous plants in temperate forests (Edwards *et al*., 2017).

Precursors of dominant families in modern temperate broadleaved deciduous forests, such as Betulaceae, Fagaceae, and Ulmaceae had existed in the high Arctic region since the Late Cretaceous in a milder and warmer environment (Zolina *et al*., 2020). They were important elements of mesothermal deciduous forests in the high Arctic region during the early Paleocene (West *et al*., 2021). However, these forests consisted of a unique combination of conifers, including Taxodiaceae, *Ginkgo*, and large‐leaved deciduous flowering plants of the Cercidiphyllaceae/Trochodendraceae, Betulaceae, Ulmaceae, Juglandaceae and Platanaceae families (Wolfe, 1985), for which we have no modern analogue at such high latitudes (Collinson, 2000; Wolfe, 1985). Their living relatives are scattered geographically in cool‐temperate, subtropical, and even tropical climates (e.g. Cercidiphyllaceae and Trochodendraceae) and maintain a relict distribution in East Asia today. By the end of the Eocene, the prolonged cooling trends and seasonally dry climate resulted in a reduction in the range of numerous thermophilic taxa and the geographic expansion of deciduous lineages accompanied by increased abundance and diversity (Fig. 1) (Butrim & Royer, 2020; Jerry & Carol, 2016; Zhang *et al*., 2022b; Utescher & Mosbrugger, 2007). Although temperate forests underwent rapid diversification and expansion from the Oligocene onward (Fig. 1) (Martinetto *et al*., 2020), the modern distribution pattern of temperate deciduous forests, characterized by distinctive elements adapted to climates with annual freezing periods, was not established until the middle Miocene (Tiffney & Manchester, 2001). A marked increase in the proportion of temperate taxa in Europe can be seen during the middle Miocene (Mijarra *et al*., 2009), while broadleaved deciduous forests in middle‐latitude eastern North America had developed at least by 13 Ma, coinciding with the onset of intense Arctic air cold fronts in winter (Wolfe, 1985). Thus, floristic modernization of temperate deciduous forests in the Northern Hemisphere unfolded progressively following the late middle Eocene climate cooling, characterized by the assembly of distinctive lineages and their geographic southward redistribution.

### (2) Development of subtropical forests in East Asia

The largest and most representative lowland subtropical evergreen broadleaved forests (EBLFs) today are found in East Asia (extending from 23° to 39° N and 95° to 141° E) (Hai *et al*., 2022; Song & Da, 2016; Tang, 2015). Their distribution is primarily determined by abundant summer rainfall and winter temperature thresholds under a monsoon climate. The keystone families of EBLFs include Fagaceae, Lauraceae, Theaceae, and Magnoliaceae, some of which also expand into the adjacent tropics, particularly the Southeast Asian and Malesian tropics (Song & Da, 2016; Tang, 2015). Many key floristic elements of the modern subtropical EBLFs are rooted in the Paleogene Laurasian “oak–laurel” forests (Shi, Xie & Li, 2014; Tang *et al*., 2016; Liu, Song & Jin, 2020), but their diversity increased rapidly from the Oligocene–Miocene boundary (OMB) through the late Miocene (Yu *et al*., 2017; Chen *et al*., 2020; Hai *et al*., 2022; Yan *et al*., 2021; Qin *et al*., 2023). A recent study based on integrative analysis of floral similarities and palaeoclimatic reconstruction revealed that subtropical EBLFs developed heterogeneously in East Asia, likely emerging initially in southern China during the middle Eocene, expanding into southwestern China from the late Eocene to early Oligocene, and establishing in central‐eastern China by the Miocene (Zhao *et al*., 2024).

The development of the subtropical EBLFs in East Asia is intrinsically linked to the evolution of the Asian monsoon under the influence of Tibetan orographic growth, which underwent reorganization through a two‐phase northward expansion (Spicer, Farnsworth & Su, 2020; Wu *et al*., 2022a). The initial phase commenced at approximately 41 Ma, with the monsoon advancing northward from tropical to southern subtropical regions (~26° N) (Wu *et al*., 2022a). The second phase began around 26 Ma, with further northward expansion to ~30–36° N, equivalent to the present‐day Asian monsoon boundary, during which a monsoon‐dominated climate progressively superseded the global wind system across central and south China (Li *et al*., 2021; Wu *et al*., 2022a). As the Asian monsoon expanded into the southern subtropical region by the middle Eocene, the previous arid/semi‐arid vegetation dominated by xerophilous taxa in southern China was replaced by the evergreen‐deciduous broadleaved mixed forest under a seasonally wet climate (Xie, Wu & Fang, 2020). By the late Eocene, mixed evergreen and deciduous forest dominated by evergreen groups of Fagaceae and Lauraceae and deciduous groups of Betulaceae, Ulmaceae and Sapindaceae thrived in southwest China (Linnemann *et al*., 2018; Sun *et al*., 2024; Wu *et al*., 2022b; Tang *et al*., 2020). These mixed forests developed under a humid subtropical climate with moderately low precipitation seasonality but colder winters than those in the region today, resulting in a higher proportion of deciduous plant components compared to modern subtropical EBLFs of the same region. Additionally, many hygrophilous conifers, such as *Sequoia*, *Metasequoia*, *Glyptostrobus*, and *Cryptomeria*, coexisted in the late Eocene to early Oligocene mixed forests but were extirpated from southwest China as the Asian monsoon intensified (Linnemann *et al*., 2017; Zhang *et al*., 2015; Sun *et al*., 2024; Ding *et al*., 2018; Wu *et al*., 2022b). Among the contemporaneous floras, such as the late Eocene Jianchuan assemblage and the early Oligocene Wenshan assemblage in southwestern China, EBLFs dominated by Fagaceae and Lauraceae developed (Tian *et al*., 2021; Gourbet *et al*., 2017), reflecting the spatial heterogeneity of plant communities that evolved under the influence of complex climatic conditions and topography in southwest China (Wu *et al*., 2022b). Despite spatial compositional variations, the consistent presence of keystone families of EBLFs, notably Fagaceae, Lauraceae, Theaceae, and Magnoliaceae (Song & Da, 2016), serve as important indicators tracing modern‐like subtropical EBLFs back to the middle to late Eocene. For example, fossil assemblages from Southwest and South China spanning from the middle Eocene to early Oligocene contained characteristic EBLF genera, such as *Cinnamomum*, *Machilus*, *Litsea*, and *Neolitsea* from the family Lauraceae and *Lithocapus*, *Quercus*, and *Castanopsis* of Fagaceae (Shi *et al*., 2014; Tang *et al*., 2016; Liu *et al*., 2020; Deng *et al*., 2020). A further substantial floristic transition from deciduous broadleaved mixed forests to predominantly EBLFs occurred during the OMB associated with the increased winter precipitation over eastern Asia as the modern monsoon system became established (Li *et al*., 2021). Phylogenetic comparative analysis supports that subtropical EBLFs rapidly assembled and diversified from the OMB through the late Miocene (Chen *et al*., 2020; Hai *et al*., 2022; Yan *et al*., 2021; Yu *et al*., 2017). Many key lineages within dominant families of EBLFs underwent significant diversification during this period, including *Lithocarpus* and the Eurasian subclade of section *Quercus* within Fagaceae (Zhou *et al*., 2022; Xiang *et al*., 2014), the tribe Perseeae (Lauraceae) (Xiao, Yan & Ge, 2022), and the Theaceae (Yu *et al*., 2017). Additionally, an accelerated emergence of evergreen habits in the *Litsea* complex (Lauraceae) occurred by the early Miocene (Qin *et al*., 2023). Beyond these featured lineages, understory components, such as the evergreen shrub *Mahonia* (Berberidaceae) and the epiphytic *Dendrobium* (Orchidaceae), arose in East Asia during the Oligocene to the early Miocene and underwent rapid radiation from the middle Miocene to the late Miocene (Chen *et al*., 2020; Xiang *et al*., 2016).

## VI. PHASE 5: THE LATE MIDDLE MIOCENE TO THE PRESENT: THE ESTABLISHMENT OF MODERN REGIONAL FLORISTIC PATTERNS

A cooler and more seasonally dry climate developed following the Mid‐Miocene Climatic Optimum (MMCO) (Steinthorsdottir *et al*., 2021; Westerhold *et al*., 2020). This climatic shift, coupled with extensive mountain building, steepened both latitudinal and vertical temperature gradients, transforming terrestrial ecosystems towards their modern configurations (Steinthorsdottir *et al*., 2021; He *et al*., 2021). This period marked the expansion of open vegetation and recent species‐level diversification across disparate ecosystems, establishing geographic diversity patterns that characterize modern biomes. Globally, many plant groups underwent significant radiations during the late Neogene in distinct ecological settings, such as succulents in deserts (Arakaki *et al*., 2011), grasses in open habitats (Spriggs, Christin & Edwards, 2014), orchids in Neotropical regions (Pérez‐Escobar *et al*., 2024), and mahoganies in tropical rainforests (Koenen *et al*., 2015).

The stronger latitudinal temperature gradient since the middle Miocene has promoted the equatorial movement and intensification of subtropical high‐pressure centres, leading to intense summer droughts and increased fire frequencies at middle–low latitudes (Rundel *et al*., 2016). These changes have triggered the development of typical mediterranean‐type vegetation (Rundel *et al*., 2016). An array of unique functional traits related to fire and drought resistance facilitated pre‐adapted or adapted lineages to colonize and diversify in this context (Onstein & Linder, 2016; Rundel *et al*., 2016). By the late Miocene, regional aridification progressively intensified in central Asia, southwest to north Africa, and Australia (Barbolini *et al*., 2020; Maestre *et al*., 2021; Caves *et al*., 2016). A notable diversity of xerophytes and succulent flowering plants has evolved in arid, desert, and semidesert habitats (Folk *et al*., 2020; Maestre *et al*., 2021), demonstrating successful adaptive strategies for surviving in these extremely harsh environments. In addition to developing unique functional traits adapted to specific biomes, certain angiosperm lineages evolved parallel strategies to cope with both freezing and arid conditions through multiple mechanisms: physiological modifications, life‐history shifts, metabolite accumulation, specialized photosynthetic pathways, and genomic organization (Wang *et al*., 2019; Folk *et al*., 2020). The exceptional ecological flexibility and adaptive capacity of angiosperms enhanced their survival and dominance in extreme environments by enabling efficient resource utilization and improved stress tolerance capabilities – adaptions that proved more challenging for other major plant groups to develop.

### (1) Expansion of grasslands, and savannas

The origin and expansion of grass‐dominated ecosystems is fundamentally intertwined with the evolutionary history of the grass family (Poaceae, Poales). Basal grasses and bambusoids are primarily forest dwelling; Pooideae and PACMAD (Panicoideae, Arundinoideae, Chloridoideae, Micrairoideae, Aristidoideae, and Danthonioideae), independently evolved adaptations to tolerate arid conditions, collectively referred to here as open‐habitat grasses (Bouchenak‐Khelladi *et al*., 2010). To date, the oldest known grass fossils are from the late Early Cretaceous (Albian, 113–101 Ma) of China (Wu, You & Li, 2018). Grasses were scarcely present in northern South America, northern Africa, and India during the latest Cretaceous and Paleocene, as evidenced by pollen and macrofossil records (Strömberg, 2011). Following successive divergences of the early Poaceae lineages over a period of ∼20 million years around the K–Pg (Huang *et al*., 2022), many tribes within Pooideae diverged from the early middle to late Eocene, with genus‐level diversification primarily occurring from the middle Miocene onwards based on phylogenetic inference (Zhang *et al*., 2022a). By the early–middle Eocene, grasses were nearly globally distributed but remained scarce within local plant communities (Graham, 1999; Strömberg, 2011). During the middle Eocene to Oligocene, fossil evidence documents a significant increase in open‐habitat grass diversity, marked by the emergence of more derived Poaceae subclades, including pooids, potential chloridoids, *Chusquea* bamboos, stipoid pooids, and the C4 PACMADs [see review by Strömberg (2011) and references therein]. The presence of open grasslands to woodlands in the late Eocene–early Oligocene is evidenced by paleosol root traces and burrowing structures typical of grasslands from northeastern Colorado (Hembree & Hasiotis, 2007), while C3‐dominated grasslands likely expanded into drier habitats by the early Miocene (Retallack, 2007). Although an increasing presence of open‐habitat grasses is documented by multiple proxies from the early to middle Miocene, grasses did not achieve widespread ecological dominance in open habitats until the middle Miocene onwards (Strömberg, 2005, 2011; Andermann *et al*., 2022). The worldwide expansion of C4‐dominated ecosystems by the late Miocene, occurring long after C4 photosynthesis evolved in grasses, points to a similar delayed ecological dominance.

The dominance of C4 grasses in tropical grasslands and savannas arises from their enhanced photosynthetic efficiency under high temperatures, arid conditions, and low atmospheric CO_2_ (Sage, 2004; Bouchenak‐Khelladi *et al*., 2009; Edwards *et al*., 2010). Multiple independent transitions from C3 to C4 photosynthesis within the PACMAD clade (with no reversals) coincided with increasing global aridification and declining atmospheric CO_2_ levels since the EOT (Bouchenak‐Khelladi *et al*., 2014; Christin *et al*., 2008; Gallaher *et al*., 2019; Edwards & Smith, 2010). The enhanced biochemical pathway of C4 grasses facilitates the assimilation of low atmospheric carbon, thus outperforming C3 grasses under hot and dry conditions at low latitudes (Huang *et al*., 2022). Carbon isotope analyses of palaeosols indicate that C4 grasses appeared in the North American Great Plains during the early Miocene (∼23 Ma) when forest‐type vegetation was dominant (Fox & Koch, 2003). Yet, the proportion of C4 biomass remained stable and moderate (12–34%) throughout the Miocene and only increased between 6.4 and 4.0 Ma, reaching modern levels by 2.5 Ma in the Great Plains (Fox & Koch, 2003). In eastern Africa, multiproxy analysis of fossil mammal sites revealed the oldest C4 grass‐dominated habitats between ~21 and 16 Ma (Peppe *et al*., 2023). By the late Miocene (*ca*. 7–8 Ma) and Pliocene, multiple lines of evidence (e.g. macrofossils, pollen, phytoliths, paleosols and stable carbon isotopes from mammalian C4 diets) show that C4‐dominated grasslands had expanded extensively across central Asia, East Africa, and North America (Strömberg, 2011; Charles‐Dominique *et al*., 2016; Pennington & Hughes, 2014; Keeley & Rundel, 2005; Retallack, 2001).

Increased seasonal aridity, decreased atmospheric CO_2_, intensified herbivore grazing pressure, and amplified fire regimes likely worked synergistically to trigger savanna expansion from the middle to late Miocene (Keeley & Rundel, 2005; Beerling & Osborne, 2006; Edwards *et al*., 2010). In particular, vegetation–fire feedback has been proposed as the main limitation to tree recruitment, allowing the expansion of C4 grasses, which in turn greatly increased ecosystem flammability in seasonally dry climate conditions (Keeley & Rundel, 2005; Beerling & Osborne, 2006; Scheiter *et al*., 2012). Additionally, the evolutionary history of fire‐adapted lineages across regions, such as *Eucalyptus* and *Banksia* in Australia (Ladiges, Udovicic & Nelson, 2003; Crisp, Cook & Steane, 2004), *Protea*, *Combretum*, and *Morella* in southern Africa (Lamont, He & Downes, 2013; Maurin *et al*., 2014), and *Mimosa*, *Andira*, *Lupinus*, and Microlicieae in the Cerrado of South America (Simon *et al*., 2009) provides evidence of their regional variation and significant within‐region diversification, coinciding with the rise to dominance of flammable C4 grasses and expansion of the savanna biome worldwide by the late Miocene (Simon *et al*., 2009; Bond, Woodward & Midgley, 2005).

### (2) Onset of Quaternary glacial cycles

The Quaternary (since 2.58 Ma) was marked by cyclical alternations between cooler, drier glacial periods and warmer, moister interglacials, which profoundly impacted sea levels, landmass area, and patterns of isolation and connectivity among populations, leading to profound consequences for species distributions and biodiversity patterns (Wang *et al*., 2021; Weigelt *et al*., 2016; Magri *et al*., 2017). The impact of Quaternary climate oscillations manifested distinctly across major plant groups and regions. Among woody species, particularly in Europe, where only a comparatively small belt of suitable (i.e. mid‐altitudinal) refugial areas was available during cold stages, both angiosperm and gymnosperm thermophilous tree species experienced significant losses (Magri *et al*., 2017; Comes & Kadereit, 1998). Angiosperms, such as *Eucommia*, *Engelhardia*, *Carya*, *Pterocarya*, *Liriodendron*, *Engelhardia* and *Liquidambar*, were eliminated from most of Europe during the Quaternary, persisting as relics in East Asia, North America, and some restricted areas in eastern Europe and the Caucasus (Magri *et al*., 2017). Gymnosperms such as *Sciadopitys*, *Cathaya*, *Tsuga*, and several Taxodiaceae were completely lost in Europe (Magri *et al*., 2017). In contrast to long‐lived trees, herbaceous plants (predominantly angiosperms) experienced relatively short life cycles, which may have enabled rapid evolutionary responses to Quaternary climate changes and enhanced their resilience (Comes & Kadereit, 1998). Many circumarctic‐alpine forbs underwent genome duplication and interspecific hybridization during glacial cycles, contributing to their survival and post‐glacial expansion (Abbott & Brochmann, 2003; Brochmann *et al*., 2004). While range contractions were common, some herbaceous species maintained widespread distributions but suffered genetic bottlenecks in recolonized populations, particularly in regions lacking suitable microrefugia (Normand *et al*., 2011; Abbott & Comes, 2004).

The Southern Hemisphere experienced spatially restricted glaciation during the Quaternary, with plant communities exhibiting higher persistence and resilience compared with the widespread displacement of species from high latitudes in the Northern Hemisphere due to extensive continental ice sheet formation (Byrne, 2008; Comes & Kadereit, 1998). For example, molecular evidence from the southern Australian flora shows the persistence and resilience of populations in localized microrefugia, contrasting with the large‐scale latitudinal migrations characteristic of Northern Hemisphere taxa (Byrne, 2008). Pollen records from New Zealand indicate that while temperate forest was reduced and fragmented during the Last Glacial Maximum, some forest elements persisted in refugial areas, particularly in northern regions (Newnham *et al*., 2013).

In mountain ranges of low to middle latitudes, Quaternary climatic oscillations drove recurrent shifts of habitats and climatic zones along elevational gradients (Flantua *et al*., 2019). These climate‐driven periodical habitat changes play dual roles in shaping species distribution and diversity patterns. On the one hand, these vertical habitat oscillations facilitated episodic biotic interchange of species, tracking their preferred temperature conditions through temporary dispersal corridors during specific climatic periods, leading to complex patterns of genetic flow among mountain populations (Ding *et al*., 2020; Rahbek *et al*., 2019). On the other hand, the repeated compression and expansion of elevation‐specific habitats stimulated speciation through cycles of population isolation and secondary contact (Flantua *et al*., 2019; Madriñán, Cortés & Richardson, 2013).

In tropical regions, the interglacial periods were characterized by a warm and moist climate and tropical forests in lowlands (as at present), whereas during glacial periods the cooler and drier climate favoured dry‐adapted forests and even open savanna‐like vegetation (Burnham & Graham, 1999; Muiruri *et al*., 2021). Repeated fragmentation and merging of geographic ranges has been proposed as a mechanism contributing to the high diversity of lowland biota by facilitating speciation through allopatric isolation and hybridization in the Neotropics (Burnham & Graham, 1999). More recent studies, however, suggest that tropical forests were in fact historically stable through glacial–interglacial cycles, with migration corridors enabling gene flow among isolated forest patches through a complex mosaic of open, semi‐open, and closed habitats (Leite *et al*., 2016; Kelley *et al*., 2024). This stability–connectivity hypothesis indicates that tropical forest resilience and sustained genetic exchange may have played a more significant role in biodiversity maintenance than previously recognized, reflecting the complex interplay between climate oscillations and vegetation dynamics during glacial–interglacial cycles.

### (3) Anthropocene

Today, the planet has entered a period in which humans act as a key force and have left an unprecedented ecological footprint. Although the Anthropocene is not currently accepted as a formal epoch in geology, it is the onset of a new transitional era, with unknown consequences on angiosperm diversity and the ecosystems that they are the foundation of (Turvey & Crees, 2019). Human activities are altering natural ecosystems and threatening numerous species, leading to an estimated one million endangered species across the tree of life (Almond, Grooten & Peterson, 2020; Nic Lughadha *et al*., 2020). Although fewer species of angiosperms are known to have gone extinct compared with other groups such as mammals and birds, their number is not insignificant (Humphreys *et al*., 2019; Antonelli *et al*., 2023): 61 tree species are currently classified as extinct or extinct in the wild by the IUCN *Red List*. Flowering plants, which constitute the majority of plant species cultivated for food, energy, or horticulture, are increasingly threatened by habitat loss, logging, trading, invasive species, and many other direct and indirect effects of human transformation of the natural environment (Díaz *et al*., 2019; Lyon *et al*., 2022). Among them, recent estimates identified around 20,000 species of trees and 4000 orchid species as being threatened with extinction (Silva *et al*., 2022; Zizka *et al*., 2021) and overall as many as 45% of all angiosperms might be threatened (Bachman *et al*., 2024). Crops and pastures are relatively recent components of the human super‐biome, leading to the rapid erosion of natural habitats, with severe loss of plant diversity (along with many other organisms). While the threat is highest in hyper‐diverse tropical regions, temperate and colder biomes are not immune to anthropogenic pressures (Silva *et al*., 2022; Díaz *et al*., 2019). Yet, unlike the contingency that led to the end‐Cretaceous mass extinction, the causes of the ongoing biodiversity crisis are predictable, and thus further biodiversity loss is preventable.

## VII. DECOUPLING BETWEEN LINEAGE ORIGINATION, DIVERSIFICATION AND THE ECOLOGICAL DOMINANCE OF ANGIOSPERMS

**Fig. 2:**
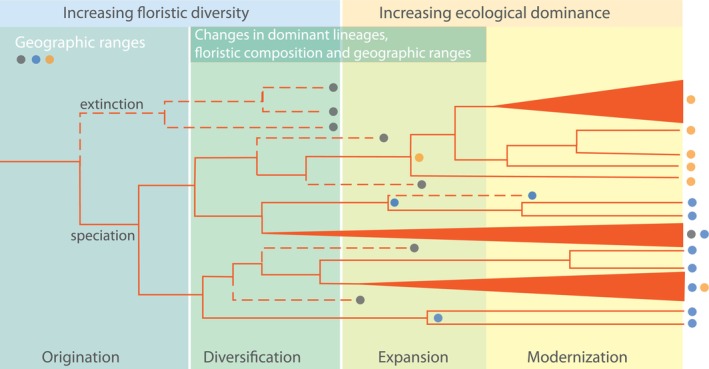
Conceptual figure showing the decoupling between lineage origination, diversification and the ecological dominance of angiosperms. Ongoing taxonomic diversification and increasing species diversity progressed alongside the expansion of angiosperm‐dominated biomes. Modernization of angiosperm‐dominated biomes occurred when key lineages achieved dominance within the floristic composition and assembled into biogeographical distributions analogous to contemporary phytogeographic patterns.

There is often a decoupling between the origination of lineages that characterize major biomes (e.g., Poaceae for grasslands, see Section (1)), lineage diversification leading to floristic diversity, and the ecological expansion and dominance of the lineage that ultimately leads to a new biome (e.g. grasslands and savanna) (Fig. 2). Although the evolutionary history of Poaceae and its association with grassland is probably the best‐known example, equivalents can be found across modern vegetation types. For example, ancestors of many major components of extant temperate deciduous angiosperm‐dominated vegetation originated in the Late Cretaceous and the Paleogene within ancient polar broadleaved deciduous forests (Zolina *et al*., 2020; West *et al*., 2021; Wolfe, 1987b). However, modernization of temperate deciduous forest did not occur until the late Eocene and through the middle Miocene concomitant with climate‐altered distribution and floristic reorganization. Likewise, the subtropical EBLFs in East Asia developed in concert with the progressive expansion of the Asian monsoon (Spicer *et al*., 2020; Wu *et al*., 2022a). Characteristic lineages of EBLFs have persisted in South China since the middle Eocene when the monsoon advanced into the southern subtropical region (Shi *et al*., 2014; Tang *et al*., 2016; Liu *et al*., 2020; Zhao *et al*., 2024). However, major floristic transitions towards the predominant EBLFs, coupled with accelerated lineage diversification, occurred from the OMB to the late Miocene, coinciding with the establishment of an Asian monsoon system comparable to present‐day patterns and its subsequent intensification (Li *et al*., 2021; Wu *et al*., 2022a). Another example is Dipterocarpaceae – a major component of pantropical forests with an intercontinental disjunct distribution, now ecologically dominant in Southeast Asian tropical rainforests. This family likely attained its ecological dominance after migrating from India to similar climatic zones in Southeast Asia following the India–Asia collision, long after its origination in tropical Africa (Khan *et al*., 2020; Bansal *et al*., 2022; Morley, 2018). While Dipterocarpaceae existed in early Maastrichtian of India (Khan *et al*., 2020; Bansal *et al*., 2022), the gymnosperm‐dominated pollen assemblages indicate that the modern equivalent of dipterocarp‐dominated forests had not yet been established (Bansal *et al*., 2022). This pattern parallels the documented delay between initial angiosperm taxonomic diversification in the middle Cretaceous and their rise to ecological dominance only in the Late Cretaceous.

## VIII. CONCLUSIONS

(1) Our review highlights five stages in the profound transformation of terrestrial ecosystems since the emergence of angiosperms in the Early Cretaceous, characterized by innovative traits and their combinations in contrast to earlier phases dominated by gymnosperms and ferns.
(2) The emergence of angiosperms around 135 Ma marked the beginning of profound evolutionary and ecological transitions in terrestrial ecosystems. Early fossil records suggest rapid geographic expansion and diversification, particularly during the Barremian and Aptian stages. This period saw angiosperms establishing new ecological niches, supported by novel reproductive and physiological traits, laying the groundwork for later dominance. By the end of the Cretaceous, angiosperms had begun to outcompete gymnosperms and ferns in many regions, although they had not yet achieved widespread ecological dominance. The mechanisms behind their initial spread and diversification are complex and involve a variety of environmental and evolutionary factors. However, the specific environmental conditions and evolutionary processes that facilitated angiosperm initial spread and diversification remain under active investigation and are not yet fully understood.
(3) Following the K–Pg boundary, angiosperms rapidly filled ecological niches and established angiosperm‐dominated rainforests in the Neotropical region. Throughout the Cenozoic, angiosperms adapted to changing climates, diversifying into the dominant floral forms found in most modern biomes. Angiosperms did not achieve ecological dominance uniformly across all biomes, underscoring the specific geographic and climatic factors that influenced the heterogeneous establishment of angiosperms in different regions, and their dynamic role in continuously reshaping terrestrial biomes in response to global environmental changes.
(4) The consistent delay between lineage origination, taxonomic diversification and subsequent ecological dominance (Fig. 2) indicates that dating of biome‐specific plant origins alone provides insufficient evidence for confident estimation of the age of their characteristic biomes. Understanding the transformation of ecosystems from early plant dominance to the proliferation of angiosperms requires not only building a repository of high‐resolution fossil records across temporal and spatial scales but also refining synthesis and analytical frameworks that integrate these data with phylogenetic reconstructions and functional trait evolution. Such advancements will provide a more comprehensive understanding of ecosystem transitions and the underlying mechanisms driving the rise of angiosperm dominance.
